# Safety and efficacy of terlipressin in acute-on-chronic liver failure with hepatorenal syndrome-acute kidney injury (HRS-AKI): a prospective cohort study

**DOI:** 10.1038/s41598-022-09505-1

**Published:** 2022-04-01

**Authors:** Anand V. Kulkarni, Sowmya Tirumalige Ravikumar, Harshvardhan Tevethia, Madhumita Premkumar, Karan Kumar, Mithun Sharma, Rajesh Gupta, Padaki Nagaraja Rao, Duvvuru Nageshwar Reddy

**Affiliations:** 1 Department of Hepatology and Liver Transplantation, Asian Institute of Gastroenterology, Hyderabad, India; 2 Department of Hepatology, PGIMER, Chandigarh, India; 3 Department of Hepatology, Mahatma Gandhi Medical College and Research Institute, Jaipur, India

**Keywords:** Hepatology, Acute kidney injury

## Abstract

Terlipressin with albumin, the recommended treatment for hepatorenal syndrome-acute kidney injury (HRS-AKI), is associated with adverse events. Furthermore, the course of AKI in patients with acute-on-chronic liver failure (ACLF) is unknown. We aimed to analyze the safety and efficacy of terlipressin infusion and AKI course in patients with ACLF. We prospectively enrolled consecutive adult patients with ACLF with HRS-AKI (satisfying EASL criteria) treated with terlipressin infusion between 14 October 2019 and 24 July 2020. The objectives were to assess the incidence of adverse events, response to terlipressin, course of HRS-AKI and predictors of mortality. A total of 116 patients were included. Twenty-one percent of patients developed adverse effects. Only 1/3rd of patients who developed adverse events were alive at day 90. Sixty-five percent of the patients responded to terlipressin. Nearly 22% developed recurrence of HRS, and 5.2% progressed to HRS-chronic kidney disease. TFS was 70.4% at day 30 and 57.8% at day 90. On multivariate stepwise Cox regression analysis terlipressin non-response (hazard ratio [HR], 3.49 [1.85–6.57]; *P* < 0.001) and MELD NA score (HR,1.12 [1.06–1.18]; *P* < 0.001) predicted mortality at day-90. Patients with ACLF who develop terlipressin related adverse events have dismal prognoses. Terlipressin non-response predicts mortality in patients with ACLF and HRS-AKI.

## Introduction

Acute kidney injury (AKI) is common in acute-on-chronic liver failure (ACLF). Organ failures, especially AKI, form the diagnostic criteria for ACLF according to the European Association for the Study of the Liver (EASL) definition^1^. Furthermore, the presence of AKI determines the outcome of patients with ACLF^2–4^. The cause of AKI in ACLF is multi-factorial^3,5^. Apart from the pre-existing profound systemic inflammation in ACLF, diuretics, sepsis, and cholestasis may impair the renal function by exacerbating hypovolemia, worsening inflammation, macrovascular dysfunction, or promoting bile salt-related direct tubular damage^3,6,7^.

Vasoconstrictor therapy (specially terlipressin) and volume expansion (with albumin) is the recommended treatment of choice for the hepatorenal syndrome (HRS)-AKI to counteract systemic arterial vasodilation and hypovolemia. However, there are limited studies assessing the role of terlipressin in patients with ACLF and HRS-AKI^8–11^.

Terlipressin is associated with adverse events in about 25–40% of patients with ACLF, and approximately 40% of those patients require treatment discontinuation^9,11^. In addition, ischemic complications and volume overload are common in patients with ACLF and high model for end-stage liver disease (MELD) score^12,13^. Continuous infusion of terlipressin leads to sustained suppression of portal pressure with a lower total dose than intermittent bolus therapy^7^. This low dose infusion protocol maintains a high mean arterial pressure (MAP) with a concomitant reduction in adverse events due to terlipressin^7,14,15^. However, a detailed evaluation of adverse events with continuous infusion and the outcomes and implications of the adverse events have not been studied in a prospective real-world study^7,16^. Furthermore, the incidence of HRS recurrence and progression to chronic kidney disease (CKD) is unknown in patients with ACLF. Here we aimed to analyze the outcomes and course of HRS-AKI in patients with ACLF treated with continuous terlipressin infusion in a real-world cohort.

## Methods

This was a prospective cohort study conducted at the Asian Institute of Gastroenterology hospital, Hyderabad, India, from 14 October 2019 and completed on 24 July 2020. We included consecutive ACLF patients aged 18–75 years treated with terlipressin infusion for HRS-AKI. We recorded the baseline clinical, demographic, and biochemical data. We excluded AKI patients treated with other vasoconstrictors (octreotide/midodrine or noradrenaline), patients with CKD, hepatocellular carcinoma (HCC), and those who refused to participate.

Written informed consent was obtained from each patient, and the study protocol conformed to the ethical guidelines of the Declaration of Helsinki. The institutional human research ethics committee (Institutional Ethics Committee-Asian Institute of Gastroenterology [IEC-AIG]) approved the study vide letter number AIG/IEC34/07.19-16 and was registered at clinical trials registry-India (CTRI/2019/10/021737). All authors had access to the study data and reviewed and approved the final manuscript.

### Objectives

The primary objective was to assess the incidence of adverse effects and its predictors. The secondary objectives were to evaluate the response to terlipressin, determine the course of AKI, assess the predictors of terlipressin non-response, and lastly, determine transplant-free survival (TFS) at day 30, 90 in patients with ACLF and HRS-AKI.

### Definitions

We included the EASL definition of ACLF^17^. Patients with HRS only were graded as ACLF I; patients with HRS and one extrarenal organ failure were graded as ACLF II, and patients who had HRS with ≥ 2 organ failures were graded as ACLF III^9^.

AKI was defined as a rise in serum creatinine (sCr) by 0.3 mg/dl or ≥ 50% rise in sCr, which is presumed or known to have occurred in the previous seven days. Patients satisfying the International Club of Ascites (ICA) criteria of HRS were classified as HRS-AKI^18^. Responders (reversal of AKI) were classified as either complete or partial responders. Complete response was defined as a reversal in the stage of AKI with a final sCr value of ≤ 0.3 mg/dL of the baseline. Partial response was defined as regression in the stage of AKI with a final sCr > 0.3 mg/dL above the baseline and non-responder if the sCr did not decrease or increased from the baseline^18^. Patients with glomerular filtration rate (GFR) < 60 ml/min per 1.73 m^2^ for three months were considered CKD patients. Sepsis was defined as per SEPSIS-3 criteria^19^.

### Management of AKI

Standard therapy was initiated, i.e., withdrawal of diuretics and volume expansion with intravenous 20% albumin infusion at a maximum dose of 1 g/kg for all patients. Terlipressin infusion was initiated at 2 mg/day in the absence of shock and response to volume expansion at 48 h, provided the renal ultrasonography, urinary protein-creatinine ratio, urine examination were normal, as per the ICA diagnostic criteria of HRS. The clinical and biochemical data after 48 h of volume expansion (at the time of initiation of terlipressin therapy) was considered baseline data and enrolled for the study. The dose of terlipressin was doubled every 48-h in case the sCr did not decrease by 25%^18^. Terlipressin 2 mg (10 ml = 1 mg) was diluted in 30 ml of normal saline and infused over 24 h at a rate of 2.1 ml/h. Electrocardiogram and echocardiography were done for all patients before initiating terlipressin. Daily clinical evaluation of the enrolled patients was done by two independent investigators (STR and AVK). Patients were followed up for 90 days to observe the recurrence of HRS and outcome (transplant-free survival).

Withdrawal of terlipressin therapy was decided based on the response to treatment and the development of adverse events. Standard care of management as per institution protocol was provided to all patients. For mild adverse events, the drug dose was reduced and monitored. Severe adverse effects requiring the withdrawal of terlipressin therapy were defined as previously suggested by Cavallin et al*.*^15^. The second line of therapy included either octreotide with midodrine or noradrenaline. The timing and modality of renal replacement therapy (RRT) was planned as per the multidisciplinary team’s (involving hepatologists, nephrologists, and intensivists) decision on a case-to-case basis.

### Statistical analysis

The data is analyzed using SPSS version 25.0 (IBM Corp, NY, USA). Descriptive statistics will be expressed as mean (standard deviation [SD]) or median (interquartile range [IQR]) for parametric or non-parametric continuous data, respectively, and number (%) for categorical data. We used the student’s t-test for comparison of means between the two groups. The categorical data are compared using Pearson’s Chi-square test (or Fisher’s exact test when required). The predictors of adverse events and terlipressin non-response are derived using stepwise multivariate logistic regression analysis involving parameters that have *P* < 0.1 on univariate logistic regression analysis and is expressed as odds ratio (OR). The predictors of mortality are derived using stepwise multivariate Cox regression analysis involving parameters that have *P* < 0.1 on univariate analysis and is expressed as hazard ratio (HR). Further, to find the different cut-off points for terlipressin non-response and mortality, receiver operating characteristic (ROC) curve analysis was also carried out. All statistical tests with *P* < 0.05 were considered significant.

### Conference presentation

The abstract was presented as a poster at IDDF 2020, Hong Kong.

## Results

During the study period, 141 patients with ACLF were diagnosed with HRS-AKI. Of them, twenty-five patients were excluded (reasons: received midodrine and octreotide-9; CKD-8; HCC-4, refusal to participate-4). Thus, a total of 116 patients with ACLF received terlipressin therapy for HRS-AKI. The mean age in the cohort was 48.31 ± 9.01 years. Ninety-four percent of patients were males. Alcohol was the most common cause of ACLF. The mean MELD-sodium (MELD NA) was 31.37 ± 7.36, and baseline sCr was 3.07 ± 1.36 mg/dl. Baseline characteristics of the included patients are shown in Table 1.

**Table 1:** Baseline characteristics of the included patients.

Variables	ACLF patients (n = 116)
Age (years)	48.31 ± 9.01
Males	109 (94%)
Etiology of liver disease (alcohol/NASH/HBV/Unknown/HCV) (n)	82/20/11/2/1
Precipitant (alcohol/HBV/DILI/HEV/sepsis/ unknown) (n)	59/10/4/1/23/19
MAP (mmHg) at inclusion	68.66 ± 6.65
Urine output at inclusion (ml/day)	825.95 ± 232.92
Hemoglobin (g/dL)	9.14 ± 1.94
Total leucocyte counts (× 10^3^ per cmm)	13.01 ± 8.36
Platelets (× 10^3^ per cmm)	130.11 ± 57.23
Total bilirubin (mg/dl)	11.5 ± 11
Serum albumin (g/dL)	2.69 ± 0.39
Blood urea (mg/dl)	101.06 ± 55.09
Serum creatinine (mg/dL)	3.07 ± 1.36
Serum sodium (meq/dL)	129.72 ± 6.71
Serum potassium (meq/dL)	4.46 ± 0.73
INR	2.04 ± 0.78
Arterial lactate	1.1 ± 0.6
Presence of sepsis at baseline	60 (51.7%)
HE at baseline (yes)	64 (55.2%)
West Haven HE grade (0,1–2,3–4)	52 (44.8%)/45 (38.8%)/19 (16.4%)
Stage of AKI (I/II/III)	17/55/44
MELD NA	31.37 ± 7.36
CLIF-C ACLF score	47.31 ± 10.39
AARC score	7.76 ± 2.43
ACLF grade (I/II/III)	62 (53.4%)/33 (28.4%)/21 (18.1%)

*EASL* European association for the study of the liver, *ACLF* acute-on-chronic liver failure, *HBV* hepatitis B virus, *DILI* drug induced liver injury, *HEV* hepatitis E virus, *HCV* hepatitis C virus, *MAP* mean arterial pressure, *INR* international normalized ratio, *HE* hepatic encephalopathy, *MELD NA* model for end-stage liver disease sodium, *CLIF-C ACLF* chronic liver failure consortium acute-on-chronic liver failure score, *AARC* APASL ACLF research consortium.

The most common source of sepsis was urinary tract infection, bloodstream infection (BSI), and spontaneous bacterial peritonitis (Supplementary Table 1). Culture was positive in 68% of patients. The most common organisms isolated were *Escherichia coli* and *Klebsiella pneumonia* (Supplementary Table 2).

### Drug therapy

The mean dose of albumin infused (per day) for the first 2 days was 44.48 ± 17.56 g/day. The mean dose of terlipressin was 2.75 ± 0.93 mg/day for a mean duration of 5.28 ± 3.51 days.

### Primary endpoint: incidence of adverse events

A total of 20.7% (95% CI, 13.76–29.2) developed adverse effects to terlipressin (Supplementary Fig. 1). Twelve percent of patients had to discontinue terlipressin due to adverse events. (Table 2) Diarrhea and abdominal pain were the most common adverse events. Of the 24 patients who developed adverse effects, 54.2% expired, 12.5% underwent liver transplantation, and 33.34% were alive at day 90 in the whole cohort (*P* = 0.03). (Supplementary Table 3) The incidence of mortality at day 90 in patients who developed ischemic adverse events was 91.7% (11/12). MELD NA score was higher in patients who developed adverse events (MELD NA adverse events group-34.46 ± 5.49 vs. no adverse events-30.57 ± 7.59; *P* = 0.02). Presence of sepsis at baseline (OR, 4.2 [1.41–12.4]; *P* = 0.01) and baseline serum bilirubin (OR, 1.07 [1.02–1.12]; *P* = 0.002) were predictors of adverse events to terlipressin on multivariate stepwise logistic regression analysis. (Table 3).

**Table 2:** Adverse effects related to terlipressin therapy.

Adverse effects	ACLF patients (n = 116)
Total	24 (20.7%)
Abdominal pain	2 (1.72%)
Diarrhea	9 (7.75%)
Abdominal pain and diarrhea	4 (3.45%)
Cyanosis	3 (2.58%)
Myocardial ischemia	1 (0.08%)
Ischemic skin necrosis	1 (0.08%)
Cyanosis + arrhythmia	1 (0.08%)
Hypertension	3 (2.5%)
Discontinuation of drug	14 (12.07%)

*EASL* European association for the study of the liver, *ACLF* acute-on-chronic liver failure.

**Table 3:** Predictors of terlipressin adverse events on univariate and multivariate stepwise logistic regression analysis.

Parameters	Univariate OR (95%CI)	*P*	Multivariate OR (95%CI)*	*P*
Age	1.009 (0.96–1.06)	0.73		
MAP at inclusion	0.98 (0.91–1.05)	0.63		
Change in MAP at day 3	1.03 (0.89–1.2)	0.68		
Presence of sepsis at baseline	3.57 (1.3–9.81)	0.01	4.2 (1.41–12.48)	0.01
Hemoglobin	1.09 (0.87–1.37)	0.44		
Total leucocyte counts	1	0.11		
Platelets	1.006 (0.99–1.01)	0.15		
Total bilirubin	1.06 (1.02–1.1)	0.003	1.07 (1.02–1.11)	0.002
Serum albumin	0.68 (0.21–2.19)	0.52		
Blood urea	1.002(0.99–1.01)	0.58		
Serum creatinine	1.32 (0.98–1.79)	0.06		
Serum sodium	0.92 (0.86–0.98)	0.02		
Serum potassium	1.5 (0.81–2.8)	0.19		
INR	1.01(0.57–1.79)	0.97		
HE at baseline	1.17 (0.47–2.9)	0.72		
MELD NA	1.08 (1.01–1.16)	0.02		
CLIF-C ACLF	1.04 (0.99–1.09)	0.06		
AARC score	1.15 (0.96–1.38)	0.11		
ACLF grade II vs 1	2.53 (0.87–7.35)	0.08		
ACLF III vs I	3.37 (1.04–10)	0.04		

*MAP* mean arterial pressure, *INR* international normalized ratio, *HE* hepatic encephalopathy, *MELD NA* model for end-stage liver disease sodium, *CLIF-C ACLF* Chronic Liver Failure Consortium acute-on-chronic liver failure score, *AARC* APASL ACLF research consortium, *ACLF* acute-on-chronic liver failure.

*Presence of sepsis at baseline, total bilirubin, baseline serum creatinine, serum sodium, MELD NA score, CLIF-C score, ACLF grade were included for multivariate analysis.

### Secondary endpoints

#### Efficacy of terlipressin in patients with ACLF and HRS-AKI and the course of AKI

**Figure 1:**
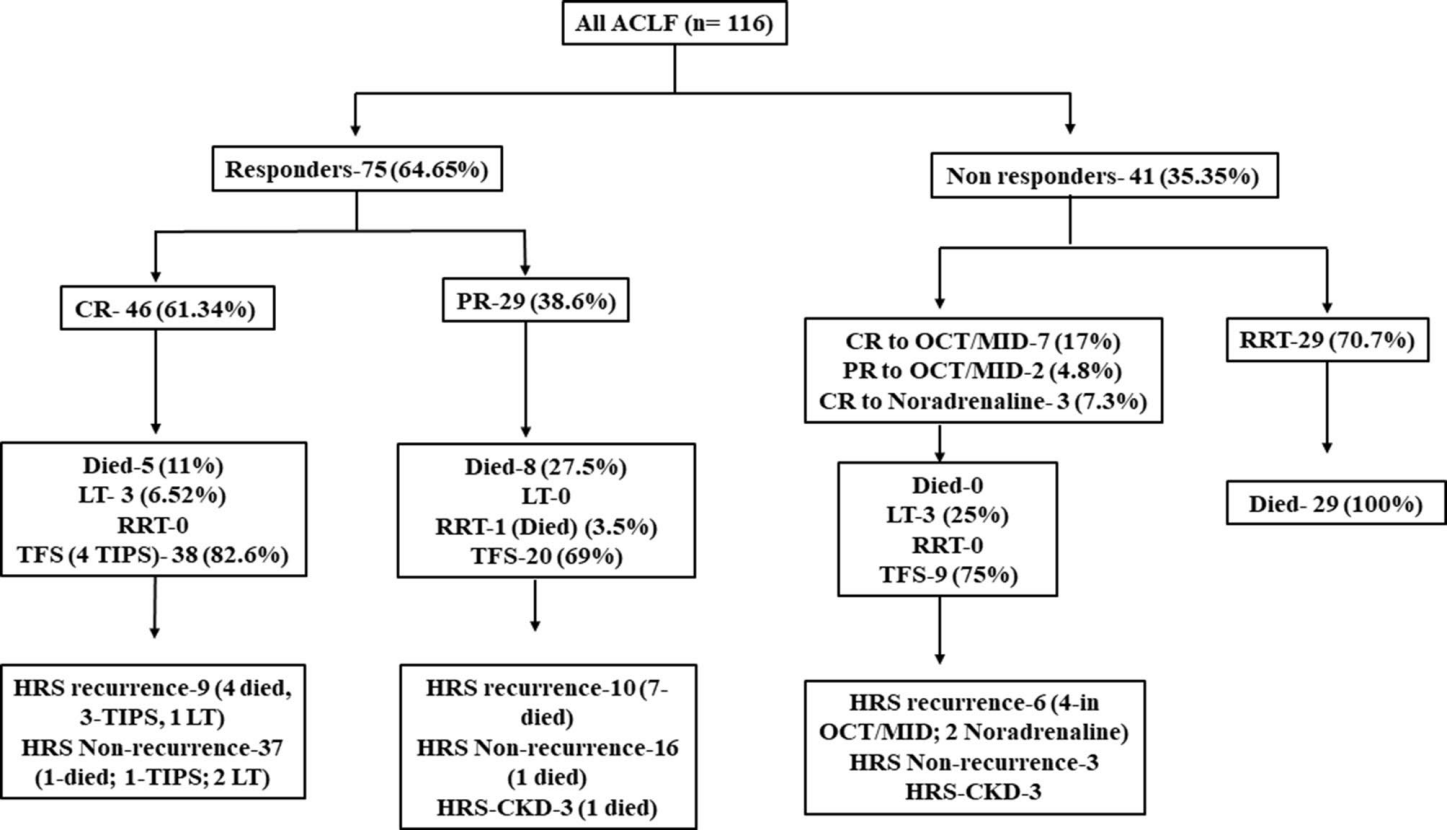
The course of HRS-AKI in the whole cohort. Of the 116 patients, 75 were responders, and 41 were non-responders. In the responder group, 46 patients achieved a complete response, and 29 patients achieved a partial response. Of the 41 non-responders, 29 required renal replacement therapy, and those requiring RRT succumbed. Nine patients of the non-responders were treated with octreotide and midodrine, and the rest were treated with noradrenaline. *HRS-AKI* hepatorenal syndrome acute kidney injury, *CR* complete response, *PR* partial response, *LT* liver transplantation, *TFS* transplant free survival, *TIPS* transjugular intrahepatic portosystemic shunt, *OCT/MID* octreotide with midodrine, *CKD* chronic kidney disease.

Sixty-five percent (95%CI, 55.23–73.3) of the patients responded to terlipressin. Complete response was noted in 39.7% (46/116) and partial response in 25% (29/116). The mean time to reversal of AKI was 4.8 ± 2.64 days. Terlipressin increased the mean arterial pressure (MAP) on day 3 by 5.23 ± 3.13 mmHg and urine output by 193.41 ± 145.65 ml/day. Twenty-six percent of patients (30/116) required RRT. All the patients requiring RRT succumbed. Nearly 22% developed recurrence of HRS, and 5.2% progressed to HRS-CKD (Fig. 1: flow of patients in the whole cohort).

#### Predictors of terlipressin non-response

On multivariate stepwise logistic regression analysis, baseline sCr (OR-2.24 [1.41–3.57]; *P* = 0.001), ACLF grade (Grade II-OR, 4.98 [1.5–16.5]; *P* = 0.009; Grade III-OR, 7.61 [1.91–30.16]; *P* = 0.004) and change in MAP at day 3 (OR, 0.73 [0.57–0.92]; *P* = 0.009) were predictors of terlipressin non-response (Table 4). Baseline sCr > 3.02 predicted terlipressin non-response with a sensitivity of 75.6%, specificity of 84% with an AUROC of 82.2 (74.1–90.4; *P* < 0.001) **(**Supplementary Fig. 2A). A change in MAP by 4.5 mmHg at day 3 predicted terlipressin response with a sensitivity of 62.7%, specificity of 67.6%, and an AUROC of 71.2 (61.5–80.9; *P* < 0.001) (Supplementary Fig. 2B).

**Table 4:** Predictors of terlipressin non-response on univariate and multivariate stepwise logistic regression analysis.

Parameters	Univariate OR (95%CI)	*P*	Multivariate OR* (95%CI)	*P*
Age	1.005 (0.96–1.04)	0.82		
MAP at inclusion	0.9 (0.83–0.97)	0.007		
Change in MAP at day 3	0.72 (0.6–0.86)	< 0.001	0.73 (0.57–0.92)	0.009
Presence of sepsis at baseline	2.89 (1.3–6.45)	0.009		
Hemoglobin	1 (0.82–1.22)	0.97		
Total leucocyte counts	1	0.001		
Platelets	1 (0.99–1.009)	0.47		
Total bilirubin	1.05 (1.01–1.08)	0.006		
Serum albumin	0.62 (0.23–1.67)	0.34		
Blood urea	1.01(1–1.01)	0.007		
Serum creatinine	2.83 (1.81–4.43)	< 0.001	2.24 (1.41–3.57)	0.001
Serum sodium	0.93 (0.87–0.99)	0.02		
Serum potassium	1.08 (0.64–1.82)	0.75		
INR	1.7 (1.03–2.8)	0.03		
HE at baseline	3.28 (1.43–7.52)	0.005		
MELD NA	1.17 (1.08–1.26)	< 0.001		
CLIF-C ACLF	1.06 (1.02–1.11)	0.004		
AARC score	1.38(1.16–1.65)	< 0.001		
ACLF grade II vs. 1	4.36 (1.69–11.21)	0.002	4.98 (1.5–16.5)	0.009
ACLF grade III vs. I	9.27 (3.03–28.33)	< 0.001	7.61 (1.91–30.16)	0.004

*OR* odds ratio, *EASL* European association for the study of the liver, *ACLF* acute-on-chronic liver failure, *MAP* mean arterial pressure, *INR* international normalized ratio, *HE* hepatic encephalopathy, *MELD NA* model for end-stage liver disease sodium, *CLIF-C ACLF* chronic liver failure consortium acute-on-chronic liver failure score, *AARC* APASL ACLF research consortium.

*Change in MAP at day 3, ACLF grade, Creatinine, Sepsis, TLC, HE, MAP day 1, blood urea, sodium, INR, Total bilirubin, AARC score, CLIF-C score, MELD NA score.

### Transplant-free survival at day 30, 90

TFS in the whole cohort was 70.4% (81/116) at day 30 and 57.8% (67/116) at day 90. Four patients underwent transjugular intrahepatic portosystemic shunt (TIPS) at day 90 for refractory ascites.

#### Predictors of mortality

On multivariate stepwise Cox regression analysis terlipressin non-response (HR, 3.49 [1.85–6.57]; *P* < 0.001) and MELD NA score (HR, 1.12 [1.06–1.18]; *P* < 0.001) predicted mortality at day-90 in the whole cohort (Table 5). Ischemic adverse events to terlipressin predicted mortality on univariate analysis but not on multivariate analysis.

**Table 5:** Predictors of mortality on univariate and multivariate stepwise cox regression analysis.

Parameters	Univariate HR (95%CI)	*P*	Multivariate HR (95%CI)	*P*
Age	0.99 (0.96–1.02)	0.51		
MAP at baseline	0.97 (0.93–1.02)	0.33		
Change in MAP at day 3	0.85 (0.76–0.96)	0.009		
Presence of sepsis at baseline	1.4 (0.79–2.48)	0.24		
Hemoglobin	1.05 (0.91–1.2)	0.48		
Total leucocyte counts	1	< 0.001		
Platelets	0.99 (0.99–1.003)	0.5		
Total bilirubin	1.03 (1.01–1.06)	0.001		
Serum albumin	0.53 (0.25–1.1)	0.09		
Blood urea	1.005(1–1.009)	0.03		
Serum creatinine	1.38 (1.18–1.62)	< 0.001		
Serum sodium	0.95 (0.91–0.99)	0.01		
Serum potassium	0.98 (0.66–1.44)	0.92		
INR	1.42 (1.06–1.9)	0.01		
HE at baseline	2.55 (1.37–4.74)	0.003		
Terlipressin non-response	5.67 (3.13–10.3)	< 0.001	3.49 (1.85–6.57)	< 0.001
Ischemic adverse events	2.95 (1.5–5.82)	0.002		
MELD NA	1.14 (1.09–1.2)	< 0.001	1.12 (1.06–1.18)	< 0.001
CLIF-C ACLF	1.03 (1.01–1.06)	0.005		
AARC score	1.34 (1.2–1.49)	< 0.001		
ACLF grade II vs. 1	3.18 (1.59–6.36)	0.001		
ACLF grade III vs. I	5.83 (2.81–12.06)	< 0.001		

*HR* hazard ratio, *EASL* European association for the study of the liver, *ACLF* acute-on-chronic liver failure, *MAP* mean arterial pressure, *INR* international normalized ratio, *HE* hepatic encephalopathy, *MELD NA* model for end-stage liver disease sodium, *CLIF-C ACLF* Chronic Liver Failure Consortium acute-on-chronic liver failure score, *AARC* APASL ACLF research consortium.

*Change in MAP at day 3, ACLF grade, terlipressin non-response, Creatinine, TLC, HE, blood urea, sodium, Total bilirubin, Albumin, AARC score, CLIF-C score, MELD NA score.

## Discussion

Terlipressin with albumin is an effective treatment for patients with ACLF and HRS-AKI^9,11^. This study has demonstrated that: (a) the overall incidence of adverse events due to terlipressin was 21% in patients with ACLF; (b) sepsis and baseline bilirubin levels predict the adverse events in patients with ACLF; (c) only 1/3rd of patients who developed adverse events were alive (without transplant) at day 90; (d) 65% of patients with ACLF responded to terlipressin; (e) the risk of recurrence of HRS was 21.5%; (f) TFS was 57.8% at day 90; (g) terlipressin non-response predicted mortality in patients with ACLF and HRS-AKI; (h) none of the patients treated with RRT survived.

The main strength of our study is the prospective collection of data on ACLF patients with HRS-AKI. Adverse events to terlipressin have been reported in earlier studies^20,21^. However, none of the studies have reported the implications and outcomes associated with it^16^. Our data is the first prospective study to report the outcomes related to adverse events due to terlipressin. MELD score was higher in patients who developed adverse events, and high MELD is known to be associated with ischemic adverse effects and, thereby, mortality^12^. Patients with adverse effects had poorer TFS. A small trial has reported fewer ischemic complications with terlipressin, even in the presence of sepsis^8^. But the study included only 18 patients with sepsis and HRS-AKI, and there was no comparator arm. Though sepsis was associated with higher adverse events, the response to terlipressin was unaltered by the presence of sepsis akin to the previous studies^8,11^. Hyperbilirubinemia is well known to be associated with poor prognosis and terlipressin response in patients with ACLF^22,23^. We noted hyperbilirubinemia to be predictive of adverse events, probably indicating the effect of cholemic injury in patients with ACLF^6^.

ACLF patients identified by EASL criteria might be diagnosed earlier and aid in prioritization for liver transplant/TIPS^24^. Terlipressin therapy is associated with a mortality benefit^11^. The adverse events of terlipressin are of great concern. Terlipressin increases the afterload and end-diastolic volume with a concurrent reduction in cardiac index^7,25^. This may unmask the pre-existing cardiac dysfunction in patients with cirrhosis and lead to volume overload and pulmonary edema, particularly if large amounts of albumin are administered concomitantly. The CONFIRM study recently highlighted the risk of pulmonary oedema in patients treated with terlipressin and albumin^13^. In contrast, we did not note any pulmonary overload due to a significantly lower dose of terlipressin and albumin than the dose used in the CONFIRM trial. Furthermore, continuous infusion of low-dose terlipressin may have prevented cardiac dysfunction in our patients.

Interestingly in our study, none of the ACLF patients treated with RRT survived at day 90. Previous studies have also reported that patients with cirrhosis requiring RRT have more than 85% mortality^26,27^. Similarly, patients with ACLF requiring RRT have high mortality. Furthermore, most of the patients included in our study had sepsis at baseline, which may have led to poor outcomes in these patients^3^.

Randomized trials with low-dose terlipressin or alternate day terlipressin therapy may be utilized to reduce the adverse effects of terlipressin in patients with ACLF. Endothelin-1/Nitric oxide ratio aid in predicting response to terlipressin therapy^28^. Serum endothelin levels, nitric oxide, lactate dehydrogenase levels may be used to monitor and predict the ischemic adverse events of terlipressin. Another unexplored area is a combination therapy of vasoconstrictors. Low-dose terlipressin, in addition to midodrine or noradrenaline, may be explored to assess the safety and efficacy in patients with ACLF and HRS-AKI.

There are certain limitations to our study. Most patients included were males with alcohol-related liver disease (ARLD). Whether males with ARLD are more prone to adverse outcomes of terlipressin in ACLF is unknown. Previous studies have also noted a higher number of male patients with ARLD developing adverse effects^12^. The use of intermittent bolus therapy was not assessed in this real-world cohort, and the results of infusion-based vs. bolus-based protocols in different study settings may skew results regarding adverse effects. Future data should also evaluate the role of cirrhotic cardiomyopathy as a confounder as this may also alter outcomes and renal response in HRS-AKI^29^.

In conclusion, our novel study described the course and outcomes of patients with ACLF and HRS-AKI. The outlying of adverse events and their effect on outcomes opens an avenue for future randomized trials, including patient selection, dosing, and alternatives.

## Supplementary Information

Supplementary Information.

## Data Availability

The datasets used during the current study are available from the corresponding author on reasonable request.

## Abbreviations

ACLF: Acute-on-chronic liver failure
AKI: Acute kidney injury
AARC: APASL ACLF research consortium
CLIF-C: Chronic Liver Failure Consortium acute-on-chronic liver failure score
EASL: European Association for the Study of the Liver
HR: Hazard ratio
HRS: Hepatorenal syndrome
IAC: International ascites club
OR: Odds ratio
sCr: Serum creatinine
TFS: Transplant-free survival
