# Supplementary material for: Safety and efficacy of terlipressin in acute-on-chronic liver failure with hepatorenal syndrome-acute kidney injury (HRS-AKI): a prospective cohort study
Source: Sci Rep. 2022 Apr 1;12:5503. doi: 10.1038/s41598-022-09505-1 (PMC8976022; doi:10.1038/s41598-022-09505-1)

**Supplementary material for the article titled:** **Safety and efficacy of terlipressin in acute-on-chronic liver failure with hepatorenal syndrome-acute kidney injury (HRS-AKI) – A prospective cohort study**

**Supplementary Table 1: Source of sepsis in the included patients**

| **Source** | **All ACLF (n=116)** |
| --- | --- |
| UTI | 23 (19.8%) |
| BSI | 10 (8.6%) |
| SBP | 8 (6.9%) |
| Pneumonia | 4 (3.4%) |
| SSTI | 4 (3.4%) |
| UTI with secondary bacteraemia | 4 (3.4%) |
| UTI +SBP | 2 (1.7%) |
| Unknown (occult) | 5 (4.3%) |

ACLF-acute-on-chronic liver failure; UTI-urinary tract infection; SBP-spontaneous bacterial peritonitis; BSI-blood stream infection; SSTI-skin and soft tissue infection

**Supplementary Table 2: Microbiological data of the included patients.**

| **Organism** | **Total number of sepsis in whole group (n=60)** |
| --- | --- |
| Culture positivity | 41/60 (68.34%) |
| E. coli | 16 (26.68%) |
| Klebsiella pneumonia | 9 (15%) |
| Acinetobacter | 1 (1.6%) |
| Candida albicans | 3 (5%) |
| Enterococcus faecalis | 4 (6.67%) |
| Enterococcus faecium | 2 (3.3%) |
| Enterobacter cloaca | 1 (1.67%) |
| MDR Klebsiella | 2 (3.3%) |
| Nocardia | 1 (1.67%) |
| Pseudomonas aeruginosa | 1 (1.67%) |
| Staphylococcus aureus | 1 (1.67%) |

E.coli-Escherichia coli; MDR-multidrug resistant.

**Supplementary Table 3: Outcome of patients who developed adverse events.**

| **Adverse effects** | **ACLF (n=116)** | **Outcome** |
| --- | --- | --- |
| Total | 24 (20.7%) | LT-3  Dead-13  Alive-8 |
| Abdominal pain | 2 (1.72%) | LT-1  Dead-1  Alive-0 |
| Diarrhea | 9 (7.75%) | LT-1  Dead-2  Alive-6 |
| Abdominal pain and diarrhea | 4 (3.45%) | LT-0  Dead-3  Alive-1 |
| Cyanosis | 3 (2.58%) | LT-0  Dead-3  Alive-0 |
| Myocardial ischemia | 1 (0.08%) | LT-1  Dead-0  Alive-0 |
| Ischemic skin necrosis | 1 (0.08%) | LT-0  Dead-1  Alive-0 |
| Cyanosis + arrhythmia | 1 (0.08%) | LT-0  Dead-1  Alive-0 |
| Hypertension | 3 (2.5%) | LT-0  Dead-2  Alive-1 |

ACLF-acute-on-chronic liver failure; LT-liver transplant.

**Supplementary figure 1: Kaplan-Meier analysis for time to adverse event.**

**
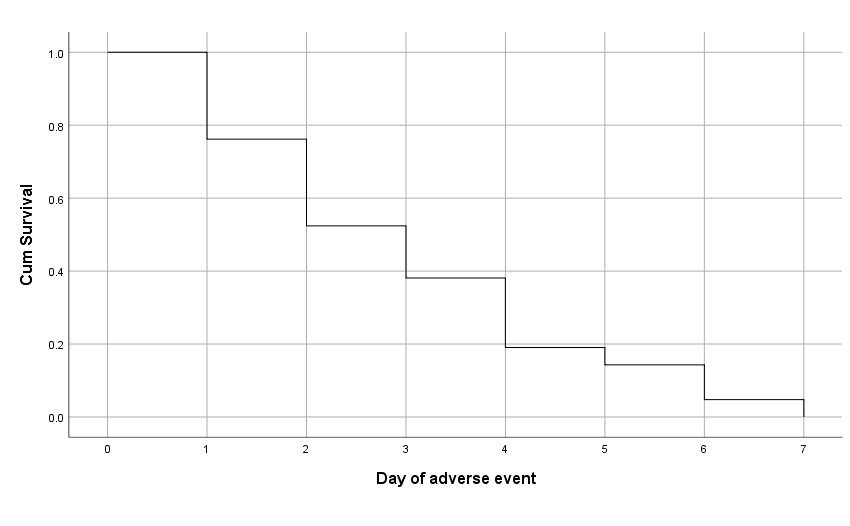
**

**Supplementary figure 2: ROC curve for baseline serum creatinine for predicting non-response (A) and mean arterial pressure for terlipressin response (B).**


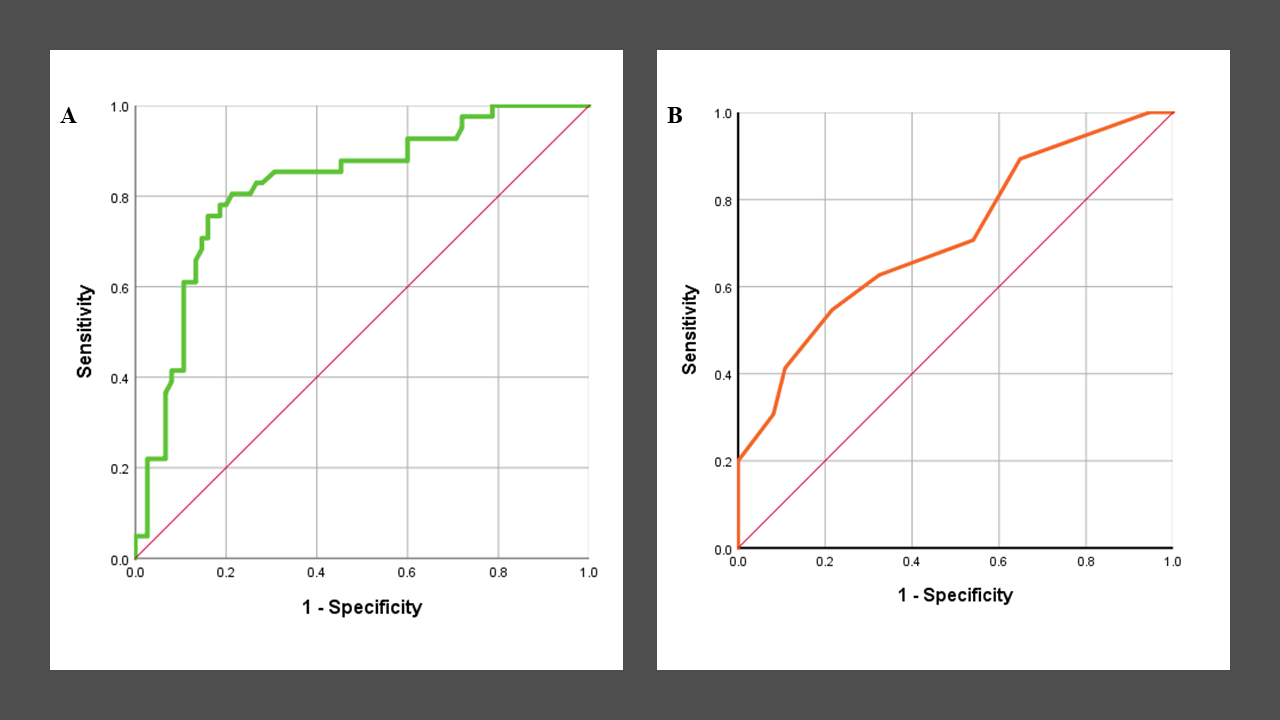

Supplement: Supplementary file 1 — Supplementary Information. [file 41598_2022_9505_MOESM1_ESM.docx]
